# Symptoms and Etiological Attribution: A Cross-Sectional Study in Mexican Outpatients with Psychosis and Their Relatives

**DOI:** 10.1155/2016/9549683

**Published:** 2016-06-16

**Authors:** Lizzette Gómez-de-Regil, Agnès Ros-Morente, Gisela Rodríguez-Hansen

**Affiliations:** ^1^Hospital Regional de Alta Especialidad de la Península de Yucatán (HRAEPY), Calle 7, No. 433 por 20 y 22, Fraccionamiento Altabrisa, 97130 Mérida, YUC, Mexico; ^2^Facultad de Educación, Psicología y Trabajo Social, Universidad de Lleida, Campus de Cappont, Avenida Estudi General 4, 25001 Lleida, Spain; ^3^Facultat de Psicologia, Universitat Autònoma de Barcelona, 08193 Bellaterra, Spain

## Abstract

This cross-sectional study aimed at identifying the most common attributions of their mental disorder in a Mexican patients who have experienced psychosis and their relatives and exploring how having experienced or not characteristic psychotic symptoms and their present clinical status might affect their etiological attributions. Past and current symptom profiles of 66 patients were as assessed with the SCID-I (Structured Clinical Interview for DSM-IV Axis I Disorders) and the PANSS (Positive and Negative Syndrome Scale), respectively. The etiological attribution of psychosis of patients (*n* = 62) and the relatives (*n* = 65) was assessed with the Angermeyer and Klusmann scale comprising 30 items into five categories: biology, personality, family, society, and esoteric. Patients and relatives attribute psychosis mainly to social factors. Relatives' attributions were not influenced by clinical profile of patients, whereas in the case of patients it was only current clinical status that showed a difference, with those in nonremission scoring higher personality and family factors. Acknowledging patients' and relatives' beliefs about mental disorders at onset and later on is particularly important in psychosis, a mental condition with severe and/or persistent symptoms, in order to promote better involvement in treatment and in consequence efficacy and recovery.

## 1. Introduction

People's beliefs about health and illness clearly affect their behavior; for instance, they can acknowledge a possible disorder and request assistance [1, 2] or agree on the benefits of a proposed treatment and then willingly adhere to it [3–5]. As a result, etiological attribution of psychosis has been a major research focus in both patients [2, 6–10] and their relatives [11–16]. How individual interprets disorder occurrence influences the pathways he/she follows when seeking help and how they deal with symptoms, with or without treatment. Beliefs about a mental disorder's etiology may also affect patient emotional response. This is particularly the case in schizophrenia spectrum disorders, the etiology of which is still unclear [17, 18]. The etiology of psychosis has been attributed to social, personality, family, biological, and even esoteric factors, and these beliefs may influence the expectations patients and their relatives have regarding prognosis and commitment to treatment [7, 8, 11].

Once under clinical treatment, patients and their relatives are usually provided with orientation about the patient's condition, possible diagnosis, treatment, and prognosis. Their previous beliefs (accurate or not) are integrated with the new information presented by mental health professionals. Schizophrenia spectrum disorder onset patterns are diverse (i.e., acute or insidious and mild to severe) as is disorder evolution, which can vary from full recovery to chronic deteriorating progression [19]. A psychotic disorder's long-term pattern can be fairly well established about five years after onset. The two to five years after the first psychotic episode, the so-called “critical period,” constitute a crucial time window in which most patients are likely to relapse and/or present residual symptoms. After this period, psychosis intensity appears to plateau and follow a more stable course [20, 21]. Attribution of psychosis in patients and in their relatives is a factor influencing pathways to care [8, 10, 16] and treatment adherence [4, 5, 14] and consequently affects prognosis and evolution. As psychosis follows its course, however, these beliefs can also transform in response to the experience of the disorder, a dynamic that has received little attention. The studies addressing beliefs about the etiology of psychosis, in patients and their relatives together, are scarce [11, 22]; the same holds true for studies in Latin America.

Using a sample of patients from a mental health hospital in Mexico, the present study aimed to (1) identify the most common factors to which patients who have experienced psychosis and their relatives attribute the mental disorder and (2) explore the possible effect of the experience of specific characteristic psychotic symptoms in the course of psychosis and their present clinical status on etiological attributions.

Understanding how patients and their relatives interpret the mental disorder is particularly important in psychosis given that symptoms are often severe and/or persistent and may require long-term treatment. Even when the individual has partially or totally recovered, the patient's and relatives' attributions can play an important role in awareness of mild signs of the disorder, either in themselves or in others, and how they proceed in response. Exploring etiological attributions in diverse samples allows cross-cultural comparisons and identification of similarities and differences. Comprehending specific etiological attributions and how they relate to the clinical profile can contribute to better interventions that target these beliefs and their management with the overall goal of improving well-being in the patient and their relatives.

## 2. Methods

This cross-sectional study received formal authorization and ethical approval from all relevant hospital committees. The protocol adhered to international [23] and national [24] ethical standards for studies with minimal risk. Participants were recruited from a public psychiatric hospital providing mental health services to anyone in need regardless of place of residence and medical insurance status.

Administrative policy at the hospital dictates that cases/files be expunged from the archives once ten years have passed from the last time the patient received attention, either as an outpatient or as an inpatient. Current clinical files were reviewed to identify patients meeting four inclusion criteria: (i) age of 16–45 years at onset; (ii) a primary current DSM-IV-TR [25] diagnosis of schizophrenia or other schizophrenia spectrum psychotic disorders; (iii) at least two years since first psychotic episode; and (iv) inhabitant of the city of Mérida, Yucatán, where the hospital is located. Exclusion criteria were (i) a DSM-IV-TR diagnosis of affective, organic, or toxic type psychosis [25]; (ii) an intellectual disability (i.e., evident limitations in intellectual functioning and adaptive day-to-day conduct, the latter is expressed as conceptual, social, and practical adaptive skills [26]) as reported by the relative and/or the patient; and (iii) insufficient contact information.

### 2.1. Participants

Clinical file review produced 161 potential cases, of which only 103 could be contacted (3 had passed away; 55 no longer lived in the area or could not be contacted). When invited to participate, patients and relatives were guaranteed confidentiality and clearly informed that their decision to participate or not would have no effect on the attention provided by the hospital or its quality and that they were free to withdraw from the study at any time. A total of 66 cases were included in the final sample; informed consent forms were signed by participants and/or their relatives with no financial compensation involved.

Over half the patients (*n* = 38, 57.6%) were female. Mean current patient age was 36.2 years (SD = 9.8) and that at psychosis onset was 29.4 years (SD = 9.6); no differences were observed by sex. Average time since onset was 6.9 years (SD = 2.1, range 3.8–14.0). Thirty-seven (56.1%) participants had an elementary and/or partial middle school education level (up to 9th grade), and the remaining 29 (43.9%) had a partial/complete middle or high school education level. None of the participants was hospitalized at the time of assessment. In terms of DSM-IV-TR [25] diagnoses, 45 patients had presented schizophrenia (25 residual, 16 paranoid, 3 disorganized, and 1 catatonic) and 21 had experienced other types of schizophrenia spectrum psychoses (9 schizoaffective, 7 delusional, 2 schizophreniform, 2 brief, and 1 unspecified).

Of the relatives (*n* = 65), 48 (72.7%) were female and mean age was 48.7 (SD = 16.5), with no differences by sex. Forty (61.5%) had elementary and/or partial middle school education levels (up to 9th grade) and the remaining 25 (38.5%) had partial/complete middle or high school education levels. Relatives included 30 (46.2%) parents, 17 (26.2%) spouses, 7 (10.8%) siblings, 6 (9.2%) offspring, and 5 (7.7%) other relatives (grandmother, aunt, nephew, mother-in-law, and sister-in-law). All relatives reported having contact with the patient at least once a week, and 58 (89.2%) lived with the patient.

### 2.2. Instruments

Patients and relatives were interviewed following module B (psychotic symptoms) of the Structured Clinical Interview for DSM-IV Axis I Disorders (SCID-I) [27] to determine whether or not characteristic symptoms of psychosis had been present at any time from onset until time of assessment. This module includes delusions (reference, persecutory, grandiose, somatic, and other delusions such as religious, guilt, jealousy, and erotomania); hallucinations (auditory, visual, tactile, and other hallucinations such as gustatory and olfactory); catatonic behavior; grossly disorganized behavior; inappropriate affect and speech; and negative symptoms. Symptom profile was complemented with information from clinical records.

Current patient clinical status was rated with the Positive and Negative Syndrome Scale (PANSS) [28, 29]. This instrument lists thirty symptoms to be scored by the interviewer from 1 (absent) to 7 (extreme); symptom dimensions are classified as positive (7 items), negative (7 items), and general (16 items). Symptomatic remission was considered as a 6-month period of simultaneous ratings of mild or less (≤3) on eight selected items: delusions; unusual thought content; hallucinatory behavior; conceptual disorganization; mannerism/posturing; blunted affect; social withdrawal; and lack of spontaneity [30].

The Angermeyer and Klusmann scale, originally tested in patient and relative samples [7, 11], was applied to participant beliefs regarding the etiology of the patient's psychosis. The authors translated the scale, which consists of a list of thirty possible causes to which participants must respond whether it is “not” (=1), “possibly” (=2), “likely” (=3), or “very likely” (=4) a cause of psychosis. Items are grouped into five etiological categories: biology, personality, family, society, and esoteric. This instrument has reported satisfactory psychometric qualities [31] in patient and relative samples [12].

### 2.3. Statistical Analysis

Descriptive statistics were generated for the patients' symptom profile and for the etiological attribution of psychosis by patients and their relatives. A series of two-way mixed (split-plot) factorial ANOVAs were used to explore the main effect of symptom profile and its interaction effect with etiological attribution. “etiological category” was entered as the intrasubject factor, with 5 levels: biology, personality, family, society, and esoteric. “Symptom profile” was considered the intersubjects factor. Patients were assigned to one of two groups for each SCID-I symptom, depending on whether they had ever experienced that symptom or not in the course of their disorder. The total number of SCID-I symptoms was generated by category (delusions, hallucinations, and other psychotic symptoms), and patients divided into two groups: those with few symptoms (<3) or many symptoms (≥3). Based on PANSS remission criteria, the patients were classified as in remission or nonremission. Tests were run independently for attribution of psychosis by patients and by their relatives.

## 3. Results

The most common symptoms experienced by patients were delusions of reference (92.4%), followed by persecutory delusions (80.3%) and auditory hallucinations (80.3%) (Table 1). As mentioned, patients were also categorized as having few or many psychotic symptoms (delusions, hallucinations, or others). When considered individually, other psychotic symptoms were not the most common (from 27.3 to 71.2%), but this category contained the most patients reporting three or more symptoms (60.6%). Negative symptoms exhibited the highest PANSS score, although overall current clinical symptoms were mild or absent since some patients could be considered as in remission. Of the single selected items for remission criteria, delusions and social withdrawal had the highest scores but remained mild (≤3).

Symptom severity prevented four patients from responding to the questionnaire; and in one case the patient could not identify a relative available for interviewing. As a result, of the 66 identified cases, 62 patients and 65 relatives reported their beliefs on etiological attribution of psychosis (Table 2). Patients and relatives concurred in pointing out “society” as the principal cause and “esoteric” as a minor cause. Application of* t*-tests indicated that the levels of attribution to each category did not differ significantly between patients and relatives. The repeated measures one-way ANOVA identified significant differences in degree of attribution between etiological categories, among both patients (*F*_(4,244)_ = 16.75; *p* ≤ 0.001) and relatives (*F*_(4,256)_ = 22.99; *p* ≤ 0.001). These differences were mainly due to attribution to esoteric factors being significantly lower in comparison to attribution to other types of factors (i.e., biology, personality, family, and society).

Overall symptom profile did not have a significant main effect on etiological category scoring, with few exceptions. Patients who had experienced auditory hallucinations (*F*_(1,60)_ = 4.080; *p* ≤ 0.05) or three or more types of delusions (*F*_(1,60)_ = 9.381; *p* ≤ 0.01) scored significantly higher in their attributions, regardless of etiological category. Relatives of those patients who had experienced other types of delusions (*F*_(1,63)_ = 7.403; *p* ≤ 0.01) or three or more types of delusions (*F*_(1,63)_ = 6.707; *p* ≤ 0.01) also scored significantly higher in their attributions, regardless of etiological category.

**Figure 1:**
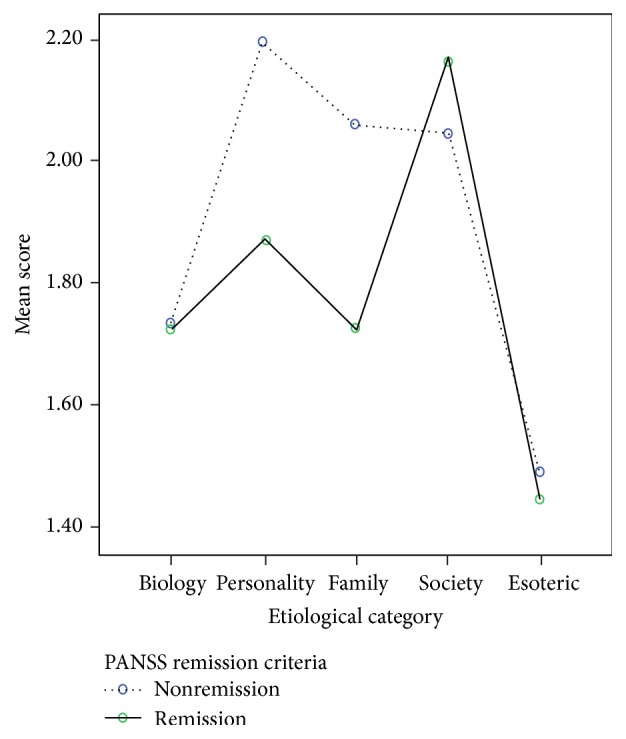
Mean attribution scores by etiological category.

No significant symptom profile × etiological category interaction was found when considering SCID-I symptoms individually or by category (*F* < 1). However, differences were observed when considering PANSS remission criteria and its interaction with etiological category (*F*_(4,240)_ = 2.428; *p* ≤ 0.05). Attribution of psychosis to biological, societal, or esoteric factors was quite similar in both patient groups, but those in nonremission scored personality (mean = 2.20) and family (mean = 2.06) factors notably higher as causes of psychosis than patients in remission (personality mean = 1.87; family mean = 1.73) (Figure 1). Among relatives, all symptom profile × etiological category interactions were not significant (*F* < 1).

## 4. Discussion

Research into the beliefs surrounding the etiology of mental disorders has gained momentum because understanding these beliefs can help to model community attitudes toward psychosis (e.g., stigmatization of patients) and identify unwarranted delays in seeking treatment [31–33]. Schizophrenia and related psychoses are characterized by a complex and often severe symptomatology. In response, interest has been focused on exploring how patients and their relatives interpret this mental condition and then consequently act when requesting and receiving treatment [5, 8]. Unlike previous studies on the subject, the present study considered the etiological attributions of psychosis of both patients and their relatives as a factor that can be influenced by the past and current profiles of psychotic symptoms.

The first study goal was to identify, in a sample from Mexico, the most common factors identified by patients and their relatives as the causes of the patient's psychotic disorder. Patients' appraisal and relatives' appraisal of etiological factors were generally similar and coincided in indicating social factors as the most important causes of psychosis and esoteric factors as the least. The attribution of psychosis mainly to social factors in the present sample concurs with prior reports from other countries such as Germany and Austria [6, 11, 13, 22], and Greece [6, 34]. However, cause attribution varies widely among reports from different countries. South-African, first-episode psychosis patients, attributed their condition mainly (49%) to spiritual causes [10]. In one study in Tunisia, patients' relatives attributed the patient's psychosis mainly (76.9%) to religious causes, such as “God's will or fate” or “God's punishment,” followed by biological causes (59.3%), and magical etiologies such witchcraft and spirit possession (47.3%) [14]. Another study in Tunisia found that 63.3% of a sample of Tunisian mothers of adolescents with first-episode psychosis attributed the child's symptomatology to spirit possession, while 77.3% considered it to be just a behavioral disorder [16].

Other studies have shown that ethnicity, rather than country of origin, has the greatest effect on etiological beliefs. A study including patients from different ethnic groups living in the UK found black and Asian individuals (all first- to third-generation immigrants) attributed their mental disorder mainly to supernatural causes, whereas white individuals, mostly (95.6%) nonmigrant British and Irish patients, attributed it mainly to individual factors [8]. In the United States, another study found that relatives of African-American patients attributed psychosis mainly to biological causes [12]. This diversity in attribution patterns highlights the need for further research across various groups; studies are still needed in community, patient, and relatives samples but also need to include different ethnic populations residing in one or more countries. Results from broader studies would allow a more thorough understanding of how patients and relatives interpret and cope with psychosis and help to design and implement targeted interventions.

The second study goal was to explore the possible effect of past and present psychotic symptom profile on etiological attributions. Whether or not a patient had experienced characteristic symptoms during the course of psychosis apparently exhibited no significant interaction with attribution to a specific etiological category in either patients or relatives. Assessment of past symptoms is retrospective, meaning that there is a possibility of interaction with attribution of psychosis in the present sample. All cases included in the study had, at some point, been under treatment at the host hospital, and patients and relatives could therefore have had some orientation about psychosis, which could partially homogenize their etiological beliefs, regardless of symptomatology. Another possibility is that individuals with similar etiological beliefs, like those in the present sample, were more likely to seek attention at a mental health hospital. Lack of significance in the results should be taken as encouragement to implement further research with more controlled methods that would allow inclusion of other possible influencing factors and more comprehensive results interpretation.

In the present sample, current clinical status had no significant interaction with etiological attribution in relatives but exhibited a significant interaction with patients' attribution. Patients in nonremission scored personality and family factors notably higher as causes than did patients in remission. Attribution to internal factors, such as personality, can expose patients to additional distress and feelings of guilt for having caused his/her mental disorder and/or for being unable to overcome it. Attribution to family can foment conflict and resentment between family members. It is important to bring these issues into clinical practice, provide patients the opportunity to openly discuss these beliefs, and then learn healthy coping skills to apply in themselves and with their families.

The present study contributes to a better understanding of how patients with psychosis and their relatives interpret this disorder. An individual's etiological beliefs about psychosis are neither right nor wrong, regardless of how much they agree or not with the current tenets of psychiatry. Systematic research has indeed generated significant amounts of data on the causes of psychosis, but comprehension of its etiology still remains limited, with a wide gap between “findings” and “understanding” [35]. Clinicians tend to emphasize and favor the biomedical aspects of psychosis in the belief that acknowledging and accepting them will promote treatment adherence and contribute to more positive outcomes [36]. This approach requires caution, however, since belief in biomedical causality seems to be linked to negative attitudes toward patients, such as perceptions of dangerousness and unpredictability, fear, and a preference for social distance [32, 37, 38]. In addition, it is unprofessional and unethical to patronize or dismiss lay beliefs; indeed, they need be respected and integrated into the process of coping with the mental disorder. Acknowledging patients' and relatives' beliefs about the mental disorder communicates practitioner interest and credibility, encouraging their involvement and increasing their commitment to treatment. It can also help clinicians to identify possible feelings of guilt, shame, or despair while treating the patient and managing the intrafamily dynamic. Allowing patients and family to openly express their beliefs about the disorder they are experiencing can be therapeutic in itself, helping them to feel understood and valued, to play an active role in an intervention model based on shared decision-making, and to have a sense of independence and efficacy [39–42]. Biomedical explanations can and should be offered, but without disregarding or disqualifying the patients' or families' etiological beliefs. In other words, mental health professionals face the challenge of integrating all the interpretations in a given case, rather than prioritizing their own over the apparently incompatible ones of the patient and his/her family [43]. It is to mental health practitioners' benefit to adopt a more eclectic treatment approach within a more open framework that will enable them to encompass local/ethnic beliefs along with traditional psychomedical treatments in therapy, since it will increase overall therapeutic efficacy [44].

The study design contains some primary limitations in terms of variable control that could influence attribution results, including previously held beliefs about mental disorders and their treatment, exposure to formal information about psychosis, the number and severity of psychotic episodes experienced, and personality traits. Nonetheless, the present results do suggest that current symptoms relate to attributions, particularly in patients. However, assuming a direct causal relationship in either direction would be inappropriate. A design including assessment at onset and follow-up at various time points would improve observation of psychosis evolution and how beliefs respond to different stages in case evolution. Finally, the studied sample was limited to inhabitants of a large urban area, leaving open the question of whether this same attribution pattern would also occur in small rural communities where social support plays an important role in individual mental health, but where esoteric beliefs are more widely held.

## 5. Conclusions

In the present results, Mexican patients with psychosis and their relatives attributed the disorder mainly to social factors. The relatives' attributions were generally not influenced by patient clinical profile. For patients, differences existed only in response to current clinical status, with those in nonremission scoring higher on personality and family factors. Acknowledging patients' and relatives' beliefs about mental disorders at onset and during treatment and follow-up needs to become a regular aspect of clinical practice to increase practitioner involvement and consequently improve treatment efficacy and recovery. This is particularly vital in mental conditions with severe and/or persistent symptoms, such as psychosis.

## Figures and Tables

**Table 1:** Patients' symptom profile (*N* = 66).

Total and percentage of patients [*n* (%)] who have ever presented characteristic psychotic symptoms as listed in the SCID-I					

Delusions	13 (19.7)^1^	Hallucinations	26 (39.4)^1^	Other psychotic symptoms	40 (60.6)^1^

Reference	61 (92.4)	Auditory	53 (80.3)	Catatonic behavior	18 (27.3)
Persecutory	53 (80.3)	Visual	38 (57.6)	Grossly disorganized behavior	25 (37.9)
Grandiose	38 (57.6)	Tactile	16 (24.2)	Inappropriate affect	19 (28.8)
Somatic	32 (48.5)	Other hallucinations	11 (16.7)	Inappropriate speech	26 (39.4)
Other delusions	45 (68.2)			Negative symptoms	47 (71.2)

Current symptomatology as assessed with the PANSS^2^ [mean (SD)]					

Positive	1.51 (0.59)	PANSS items proposed as remission criteria^3^: 41 (62.1%) patients in remission			
Negative	1.78 (0.84)	Delusions	2.02 (1.38)	Mannerisms/posturing	1.24 (0.84)
General	1.63 (0.49)	Unusual thought content	1.52 (0.93)	Blunted affect	1.82 (1.14)
Total	1.64 (0.53)	Hallucinatory behavior	1.44 (0.91)	Social withdrawal	2.27 (1.60)
		Conceptual disorganization	1.64 (1.16)	Lack of spontaneity	1.55 (1.13)

^1^Number and percentage of patients who had experienced many symptoms (≥3) of the category.

^2^Mean scores are reported between the ranges of 1 (absent) and 7 (severe).

^3^Simultaneous ratings of mild or less (≤3) on all required items.

**Table 2:** Etiological attribution of psychotic illness reported by patients and their relatives.

		Patients (*n* = 62)	Relatives (*n* = 65)
		Mean (SD)	Mean (SD)
*Biology*			
Disturbance of brain biochemistry, hereditary factors, birth trauma, brain injury, organic disease external to brain, infectious brain disease		1.73 (0.58)	1.84 (0.65)

*Personality*			
Avoidance of everyday life problems, failure in life, lack of willpower, too bright or intelligent, too ambitious, drug/alcohol abuse		1.98 (0.62)	1.82 (0.61)

*Family*			
Broken home, lack of parental love, parent attitude hostile-rejecting, father too severe, overprotective mother, parental expectations too high		1.84 (0.75)	1.93 (0.79)

*Society*			
Stressful life events, constant strain in school/job, troubles in marriage/partnership, society, loneliness, influence of bad friends		2.12 (0.67)	2.07 (0.73)

*Esoteric*			
Possession by evil spirits, lack of vitamins, punishment by God, unfavorable horoscope, radiation, environmental pollution		1.46 (0.52)	1.36 (0.46)

Significant post hoc results in patients		Significant post hoc results in relatives	

Biology < personality^*∗*^	Family < society^*∗*^	Biology > esoteric^*∗∗∗*^	Family > esoteric^*∗∗∗*^
Biology < society^*∗∗∗*^	Family > esoteric^*∗∗*^	Personality < society^*∗∗*^	Society > esoteric^*∗∗∗*^
Biology > esoteric^*∗*^	Society > esoteric^*∗∗∗*^	Personality > esoteric^*∗∗∗*^	
Personality > esoteric^*∗∗∗*^			

Individual item scores: 1 (not a cause), 2 (possibly a cause), 3 (likely a cause), 4 (very likely a cause).

^*∗*^ *p* ≤ 0.05, ^*∗∗*^ *p* ≤ 0.01, and ^*∗∗∗*^ *p* ≤ 0.001.
